# Study on the effect of size on InGaN red micro-LEDs

**DOI:** 10.1038/s41598-022-05370-0

**Published:** 2022-01-25

**Authors:** Ray-Hua Horng, Chun-Xin Ye, Po-Wei Chen, Daisuke Iida, Kazuhiro Ohkawa, Yuh-Renn Wu, Dong-Sing Wuu

**Affiliations:** 1 Institute of Electronics, National Yang Ming Chiao Tung University, Hsinchu, 30010 Taiwan, ROC; 2 Department of Materials Science and Engineering, National Chung Hsing University, Taichung, 40227 Taiwan, ROC; 3 Computer, Electrical and Mathematical Sciences and Engineering (CEMSE) Division, King Abdullah University of Science and Technology (KAUST), Thuwal, 23955‑6900 Saudi Arabia; 4 Graduate Institute of Photonics and Optoelectronics, National Taiwan University, Taipei, 10617 Taiwan, ROC; 5 Department of Applied Materials and Optoelectronic Engineering, National Chi Nan University, Nantou, 54561 Taiwan, ROC

**Keywords:** Nanoscience and technology, Optics and photonics

## Abstract

In this research, five sizes (100 × 100, 75 × 75, 50 × 50, 25 × 25, 10 × 10 µm^2^) of InGaN red micro-light emitting diode (LED) dies are produced using laser-based direct writing and maskless technology. It is observed that with increasing injection current, the smaller the size of the micro-LED, the more obvious the blue shift of the emission wavelength. When the injection current is increased from 0.1 to 1 mA, the emission wavelength of the 10 × 10 μm^2^ micro-LED is shifted from 617.15 to 576.87 nm. The obvious blue shift is attributed to the stress release and high current density injection. Moreover, the output power density is very similar for smaller chip micro-LEDs at the same injection current density. This behavior is different from AlGaInP micro-LEDs. The sidewall defect is more easily repaired by passivation, which is similar to the behavior of blue micro-LEDs. The results indicate that the red InGaN epilayer structure provides an opportunity to realize the full color LEDs fabricated by GaN-based LEDs.

## Introduction

Solid-state light emitting diodes (LEDs) have the advantages of self-luminescence, high brightness, and good stability. Compared with organic LEDs (OLEDs), they offer higher resolution, contrast, and a longer life, thus gaining immense popularity^1,2^. To achieve high resolution, the size of the micro-LEDs (μ-LEDs) must be reduced so that more pixels can be placed in a unit display area. Up to now, blue GaN-based and red AlGaInP-based LEDs have been fabricated into μ-LEDs with dimensions smaller than 100 × 100 μm^2^. Chip size is found to affect the performance of μ-LEDs. The decrease in the external quantum efficiency (EQE) of μ-LEDs with size has been widely studied and reported on^3,4^. The increasing ratio of surface area to volume for the device results in various non-radiative losses at the device edge. The defects at the sidewall are caused by an inductively coupled plasma (ICP) etching process, which led to the increased Shockley–Read–Hall (SRH) non-radiative recombination. These defects result in lower EQE of μ-LEDs with smaller diameter^4,5^. Although sidewall passivation can improve the injection current efficiency and EQE, AlGaInP μ-LEDs typically suffer from much stronger size-dependent efficiency reduction owing to their high surface recombination velocities, longer carrier diffusion lengths^6,7^, and limited improvements through sidewall passivation^8^. Recently, InGaN-based red μ-LEDs have been successfully grown by metal–organic chemical vapor deposition (MOCVD) on Ga_2_O_3_, sapphire, and Si substrates^9–11^. Most studies on the red light InGaN epilayer focused on the characteristics of its epitaxial technology, structure, and emission wavelength. To increase the optical power output, the thickness of the undoped GaN layer and the n-GaN layer is increased, which results in the emission wavelength and EQE of the red InGaN LED (@20 mA) to be 633 nm and 1.6%, respectively^12,13^. Although ultra-small (< 10 μm) 632 nm red InGaN μ-LEDs with useful on-wafer EQE (> 0.2%) has been published^14^, the chip size effect on the InGaN μ-LED’s performance has not been studied. In this work, the red light InGaN structure was used to fabricate μ-LEDs with dimensions of 100 × 100, 75 × 75, 50 × 50, 25 × 25, and 10 × 10 μm^2^ for the chip size effect study. The optoelectronic characteristics were analyzed through the current and voltage relation, electroluminescence (EL) spectra, and light output power density as a function of the chip size of μ-LEDs.

## Results and discussion

**Figure 1:**
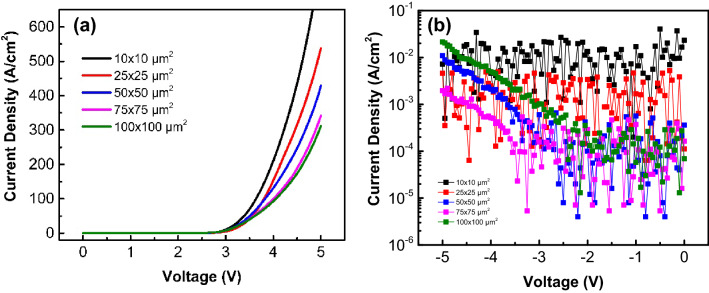
(**a**) Forward current density–voltage (J–V) characteristic for all chips and (**b**) leakage behavior for μ-LEDs with different sizes.

Figure 1a shows the forward current density–voltage (J–V) characteristic for all chips. The turn-on voltage of μ-LEDs from large to small chips is 3.05, 3.06, 3.04, 3.13, and 2.99 V, respectively, for an injected current density of approximately 10 A/cm^2^ (corresponding 1 mA for 10 × 10 μm^2^). At 4 V, the current density of μ-LEDs from large to small chips is 92.7, 99.8, 131, 154, and 210 A/cm^2^, respectively. Clearly, the current density increases as the size decreases. When the μ-LED light-emitting area is reduced, the volume and resistivity also reduce. Under the same current density, the forward voltage decreases as the chip size is reduced. Moreover, under the same bias voltage, a small-sized μ-LED will have a larger current density.

The main issue is the leakage current for μ-LEDs. The leakage behavior for μ-LEDs with different sizes is shown in Fig. 1b. It was previously reported that the leakage current is related to the sidewall^5^. The relationship between the sidewall surface area and the total surface area of ​​the μ-LED is shown in Table 1. First, the same number of defects per unit sidewall surface area is assumed. As the size is reduced, the sidewall surface area and the number of sidewall defects also decreases; nevertheless, the ratio of sidewall surface area to the total surface area increases. This suggests that the number of defects per unit total area increases and the effect of sidewall defects also increases for small-sized μ-LEDs.

**Table 1:** Comparison of total surface area and side wall surface area of ​​different sizes of μ-LEDs.

μ-LED dimensions (μm^2^)	Mesa area (μm^2^)	Sidewall area (μm^2^)*^a^	Sidewall surface ratio*^b^
10 × 10	100	20.6	0.171
25 × 25	625	51.5	0.076
50 × 50	2500	103	0.040
75 × 75	5625	154.5	0.027
100 × 100	10,000	206	0.020

*^a^Mesa depth = 515 nm.

*^b^Sidewall surface ratio = sidewall area/total surface area.

It is found that the leakage current density increases as the size decreases under a reverse voltage less than 3 V. The leakage density of μ-LEDs with the smallest dimensions was the highest. This indicates that the leakage current could be caused by sidewall defects. Moreover, it could also be caused the largest electric field from the electrode to the edge (shortest distance = 3.5 μm for μ-LED with 10 × 10 μm^2^) as the reverse voltage less than 3 V. The electric field (@ − 3 V) from the electrode to the edge was 2.3 × 10^3^, 4.0 × 10^3^, 6.0 × 10^3^, 4.8 × 10^3^, and 8.5 × 10^3^ V/cm of μ-LEDs from large to small chip size. It is worthy to mention that the leakage current density of μ-LEDs from large to small chip size is 2.14 × 10^−2^, 1.96 × 10^−3^, 1.09 × 10^−2^, 4.59 × 10^−3^, and 7.2 × 10^−3^ A/cm^2^ for a reverse voltage of 5 V. As the reverse voltage increases, the leakage increases more obviously for larger chip size, especial for the μ-LED with 100 × 100 μm^2^. This suggests that not only is the leakage current relative to the sidewall passivation and the lateral electric field, the defects distributed on the surface could also affect the leakage current density. The large-sized LEDs cover a large area. It causes the leakage current density of large-sized μ-LEDs (100 × 100, 75 × 75, and 50 × 50 μm^2^) to increase and to be close to, and even higher than, those of small-sized μ-LEDs (20 × 20 and 10 × 10 μm^2^). Because there exist many defects in the InGaN epilayers^12,15^, this could also cover more defects. This causes leakage as the reverse bias increases.

**Figure 2:**
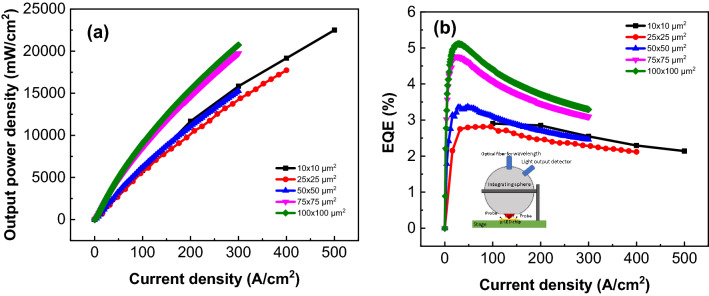
(**a**) Optical output power densities and (**b**) EQE as function of injection current densities for all chip size μ-LEDs. The setup of on-wafer EQE measurement shown in the inset of (**b**).

Figure 2a shows the optical output power density as a function of the injection current density. As the injected current increases, the optical output power increases for all the chips. It was found that there were two groups. One group is for the larger chips with areas of 100 × 100 and 75 × 75 μm^2^. The other group is for chip sizes with areas 50 × 50, 25 × 25, and 10 × 10 μm^2^. If the output power density is affected by the sidewall, the smallest chip μ-LEDs should show the lowest power density. As shown in Table 1, the μ-LED with an area of 10 × 10 μm^2^ presents the largest sidewall surface ratio. If leakage is the main issue affecting the output power, the short distance between the p-electrode and the edge could be an important parameter. As shown in Fig. 5b, the short distance between the p-electrode and the edge is 12.5, 7.5, 5, 6.3, and 3.5 μm for all chips and sizes in order. Moreover, at the same injection current density, the distance from the electrode to the edge is only 3.5 μm for the smallest size chip. Considering the sidewall surface ratio and the distance to the edge, the smallest dimension μ-LEDs would obtain the smallest output power density. However, for the 10 × 10 μm^2^ μ-LEDs shown in Fig. 2a, this does not produce the smallest output power density. Evidently, the passivation layer provides the sidewall protection from leakage. This causes the output power density to be very similar for smaller μ-LEDs. This is different from the behavior of AlGaInP μ-LEDs. Obviously, the sidewall defect is more easily repaired by passivation. This is similar to the behavior of blue μ-LEDs. Larger chips (100 × 100 and 75 × 75 μm^2^) show higher output power density than smaller chips (50 × 50, 25 × 25 and 10 × 10 μm^2^). This might be attributed from the fact that larger chips contribute to light reflection from the bottom.

Figure 2b shows the EQE as a function of injection current densities. The maximum EQE is 5.11, 4.75, 3.36, 2.81 and 2.91% corresponding to the chip size in order. Note that the maximum EQE occurs at almost the same current density, except the 10 × 10 um^2^ chip. It is due to the output power is too small to measure as the low injecting current density for the 10 × 10 um^2^ chip. Moreover, it has been reported that the maximum EQE is shifted to higher current densities as the chip size decreases for the AlGaInP uLEDs. The shift of maximum EQE was related to leakage current and/or an increased Shockley–Read–Hall (SRH) non-radiative recombination at sidewall defects in the smaller geometries^14,16^, although the sidewall had been passivated. However, the phenomena does not be observed in the red InGaN uLEDs. The obtained results were consistent with that in Fig. 2a, i.e., the sidewall defects can be repaired by passivation. On the other hand, the droop behavior was more alleviative for the small size uLEDs. It could be due to the fact that there exist more defects or phase separation for the uLEDs with larger size. These defects could trap the carriers and reduce the EQE. As concerning this point, it has been demonstrate by TEM measurement^12^. Furthermore, it was found that the EQE of the uLEDs with10 × 10 um^2^ was higher than those of uLED with 25 × 25 um^2^ and 50 × 50 um^2^. For smaller mesa size, the contribution of light coupled through the mesa edge compared to the surface becomes larger. This causes an increase of the external quantum efficiency (EQE) by light extraction. It was worthy to mention although the epilayer structure was the same with Refs.^12,14^, the obtained EQE in this work is higher than those of Refs.^12,14^. It could be due to the different device fabrication parameters, geometry of the metallic contacts, mesa outline and chip sizes.

**Figure 3:**
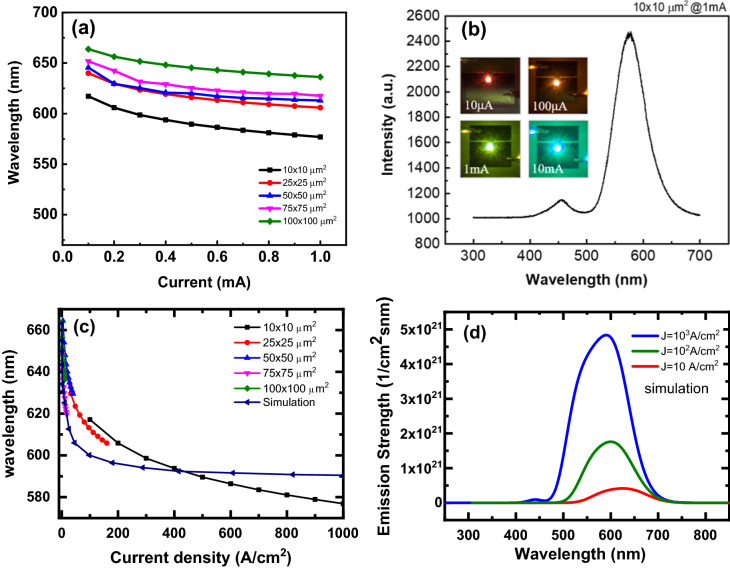
(**a**) Wavelength as a function of current of red μ-LEDs of different sizes under driving currents from 0.1 to 1 mA and (**b**) EL spectrum for the μ-LED with area 10 × 10 μm^2^ for an injection current of 1 mA. (**c**) Wavelength shift versus current density for both experimental and simulation results and (**d**) Simulation emission spectra under different current density.

Figure 3 shows the wavelength of μ-LEDs with different sizes as a function of current. The wavelength decreases as the current increases, exhibiting blue shift behavior. It is well known that the blue shift phenomenon in blue and green InGaN-based LEDs grown on c-plane sapphire due to the InGaN QWs is caused by the screening of the piezoelectric field and band filling of the localized state. The same behavior is also exhibited in the InGaN μ-LEDs with high In composition^13–18^. Moreover, the wavelength decreases as the chip size is reduced. This can be attributed to several possible reasons. The first is stress release in small dimensional LEDs. Second, smaller-sized chips have larger current densities, which leads to screening of QCSE and also band filling effects. To verify this, we applied a 1D Poisson and drift–diffusion solver (1D-DDCC) developed by Prof. Wu’s lab in NTU^19,20^. With the same structure as shown in Fig. 1, we can simulate the band bending, confined energy, and emission spectrum at different densities. Figure 3a indicates that at a driving current of 0.1 mA, the emission wavelengths from large to small are 663.78, 651.76, 645.51, 639.97, and 617.15 nm in order. Furthermore, the emission wavelengths from large to small are 636.24, 617.55, 612.98, 605.87, and 576.87 nm in order as a driving current increasing to 1 mA. Obviously, the μ-LED with 10 × 10 um^2^ did not emit the red color wavelength as the injection current was higher than 0. 1 mA. The current density versus chip size as shown in Fig. 3c reveals a trend with small variation for different chip sizes. To understand the mechanism for this large range of blue shift, 1D simulation was performed for different current densities. It shows that blue shift from 660 nm (0.1 A/cm^2^) to 590 nm (1 kA/cm^2^) is possible. This blue shift is caused by (1) screening of QCSE and (2) band filling effects. Our calculations show that blue shift from 660 to 620 nm is mainly due to the screening of QCSE. After 10 A/cm^2^, the band filling effect becomes stronger and the emission spectrum widens as shown in Fig. 3d. The trend of blue shift matches better with larger chip size. For smaller chip size such as 25 and 10 µm, the trend is slightly different. The discrepancy may be due to several possible reasons: (1) the current crowding effect is different for different chip sizes. Hence, the red shift may not occur for different current densities in the QW as predicted by 1D simulations; (2) for the high indium QW, a large random alloy fluctuation or indium segregation is expected. Hence, the filling of states may not be the same as the ideal cases; (3) although the compressive InGaN is relaxed to 0.38 GPa^12^, the top multi-QWs of InGaN still suffer from compressive strain. Hence, the wavelength shift results from a combination of the screening of QCSE, quantum effect, and band filling. This is a complex phenomenon and requires further study. By contrast, there exist extra emission spectra at 380 and 450 nm for the smallest μ-LEDs for high injection current density, as shown in Fig. 3b. Obviously, the overflow current and band filling phenomena occur in the red InGaN μ-LEDs. The inset of Fig. 3b also shows the emission images of the 10 × 10 μm^2^ μ-LED injected at the current from 0.1 to 10 mA. In the simulation, if the leakage path is strong in the epilayer, with a higher density of tail stats in the simulation, we also observe that some holes will reach the blue QWs for current density larger than 10^3^ A/cm^2^ and an emission of blue QWs will be observed. These leakage paths may come from dislocation line and Vpits^12^.

## Conclusion

In this study, five InGaN red light μ-LEDs with areas 100 × 100, 75 × 75, 50 × 50, 25 × 25, and 10 × 10 μm^2^ were successfully produced. The leakage current density increases as the size decreases under a reverse voltage less than 3 V. As the reverse voltage increases (> 3 V), the leakage increases more obviously for larger chip size, especial for the μ-LED with 100 × 100 μm^2^. This suggests that not only is the leakage current relative to the sidewall passivation and the lateral electric field, the defects distributed on the surface could also affect the leakage current density. The optical output power increases for all the chips with the injected current. When the injection current was 0.1 mA, the emission wavelengths of μ-LEDs from large to small were 663.78, 651.76, 645.51, 639.97, and 617.15 nm. As the size of the μ-LED decreased, the injection current of the small-sized μ-LED increased, the blue shift of the emission wavelength caused by the band filling effect became more obvious. In order to maintain the red color emission (wavelength > 615 nm) for the 10 × 10 μm^2^, it should be operated current less than 100 μA. The results indicate that InGaN materials can successfully produce red μ-LEDs with a minimum size of 10 × 10 μm^2^, which has the potential to become a full-color display of InGaN structure with blue and green light for μ-LEDs display applications.

## Experimental

**Figure 4:**
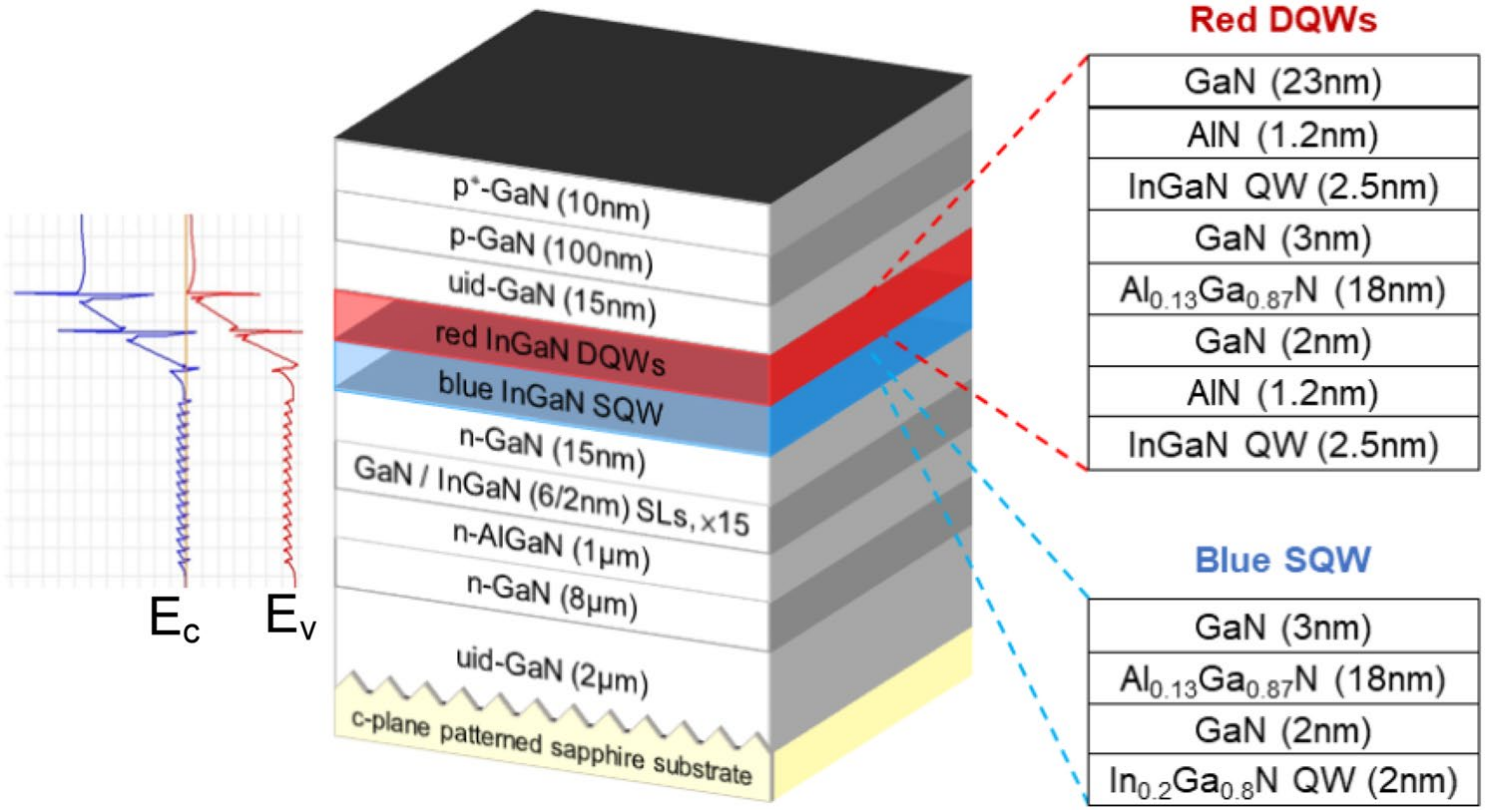
Schematic diagram of red InGaN µ-LED epitaxial structure and the corresponding energy bandgap.

The red InGaN μ-LED structure was grown on the c-plane patterned sapphire substrate by MOCVD. First, an undoped thick GaN layer with 2 μm thickness was grown to reduce the residual stress and improve the quality of the following InGaN quantum well (QW). Then, the Si-doped n-type GaN layer with 8 μm thickness and 15 pairs of GaN(6 nm)/InGaN(2 nm) superlattice layers were grown for stress engineering. Subsequently, a blue InGaN single-layer QW with a small amount of In and 2 pairs of red InGaN QWs with a large amount of In, and p-type GaN doped with Mg were sequentially grown. Figure 4 shows the details of the epitaxial layer structure as well as the band diagram. The crystal properties and microstructure examined by atomic force microscopy, scanning electron microscope and transmission electron microscope have been studied and published^12^. After, the epilayer was cleaned by acetone, isopropanol, and deionized water, an indium tin oxide (ITO) film with 280 nm thickness was deposited on p-GaN using an electron gun evaporation system as the current spread layer. The samples were annealed at 525 °C for 20 min to make the ITO and the p-type GaN layer present Ohmic contact.

**Figure 5:**
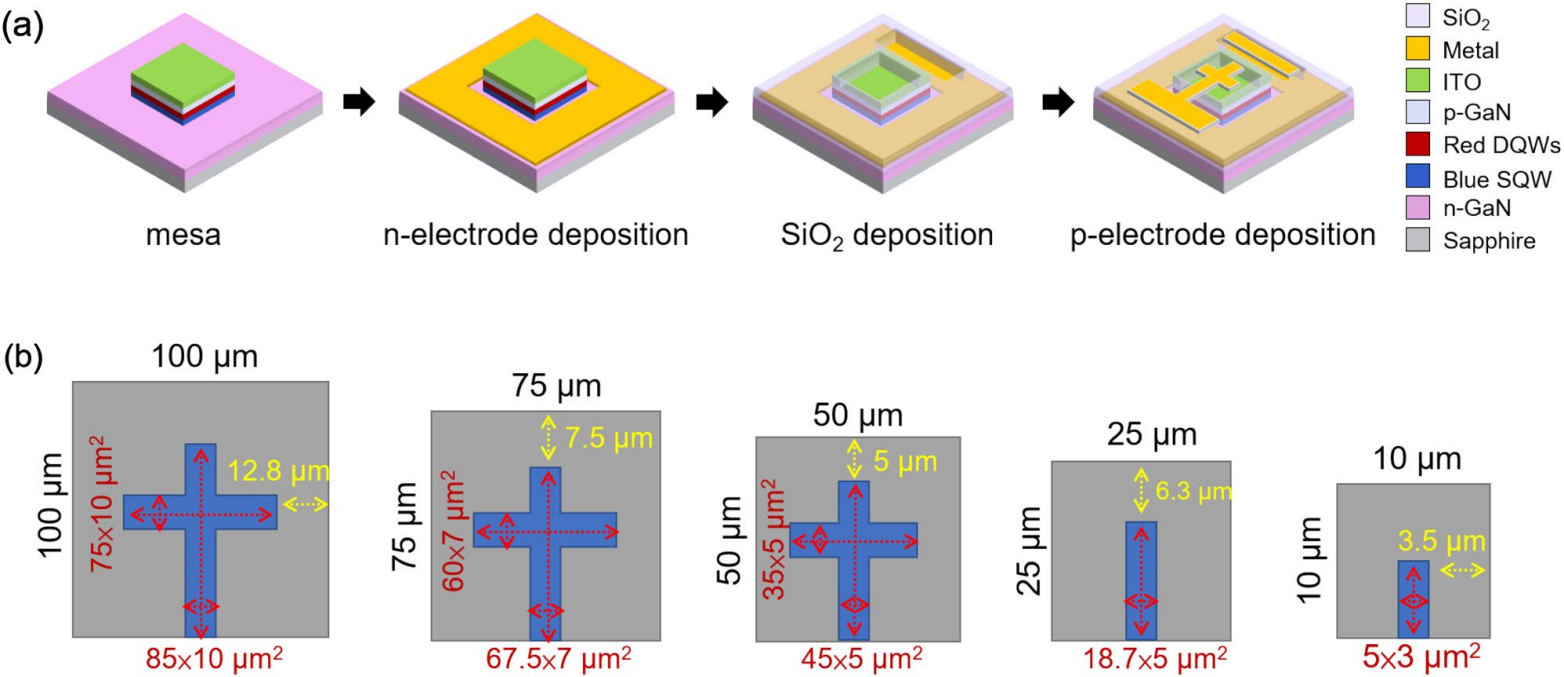
(**a**) Fabrication flowchart of μ-LEDs and (**b**) all the p-electrode dimensions of μLEDs.

In this study, laser direct writing (Heidelberg Instruments, MLA-150) exposure technology was used for the pattern process without mask, and μ-LEDs of different sizes (i.e., 100 × 100, 75 × 75, 50 × 50, 25 × 25, and 10 × 10 μm^2^) were fabricated on the same epitaxial wafer. After the mesa pattern, the epilayer was etched to the n-GaN layer by an inductively coupled plasma reactive ion etching (ICP-RIE) system. Then, Ti/Al/Ti/Au/Cr multilayer metals were deposited on the n-GaN as the n-contact electrode, and 525-nm-thick SiO_2_ was grown by plasma enhanced chemical vapor deposition (PECVD) as the passivation layer to repair the dry etching damage on the sidewalls of the μ-LEDs and the insulating layer to isolate the anode and cathode metal electrodes. Finally, Cr/Al/Ti/Au multilayer metals with a thickness of approximately 1 μm were deposited as the p contact electrode by an electron gun evaporation system. Details on the fabrication flowchart of μ-LEDs are shown in Fig. 5a. It is important to mention that the p-electrode contact pads for all sizes were extended to the mesa area for easy measurement. All the p-electrode dimensions are shown in Fig. 5b; they cover about 15% of the emission area.

After processing, the current–voltage (*I–V*) and output power characteristics of these µLEDs were measured at room temperature. The EQE was obtained by on-wafer measurements which the integrating sphere covers the wafer and light was collected through the surface, shown in the inset of Fig. 2b. Due to the limited collection angle and lack of light extraction enhancements (without encapsulation by epoxy), the measured EQE values could be lower compared to values obtained if the devices were encapsulated and the total extracted light collected in an integrating sphere. Even though, the EQE was calibrated using a TO-Can packaged red GaInN LED with 300 µm × 300 µm (with the same fabrication process and epilayer) without encapsulated by epoxy and was measured in an integrating sphere. All data were averaged using 20 µLEDs devices.
